# Time-Resolved Gene Expression Analysis Monitors the Regulation of Inflammatory Mediators and Attenuation of Adaptive Immune Response by Vitamin D

**DOI:** 10.3390/ijms23020911

**Published:** 2022-01-14

**Authors:** Andrea Hanel, Carsten Carlberg

**Affiliations:** Institute of Biomedicine, School of Medicine, University of Eastern Finland, FI-70210 Kuopio, Finland; andrea.hanel@uef.fi

**Keywords:** vitamin D, 1α,25-dihydroxyvitamin D_3_, transcriptome, PBMCs, vitamin D target genes, inflammatory response, adaptive immune system

## Abstract

Peripheral blood mononuclear cells (PBMCs) belong to the innate and adaptive immune system and are highly sensitive and responsive to changes in their systemic environment. In this study, we focused on the time course of transcriptional changes in freshly isolated human PBMCs 4, 8, 24 and 48 h after onset of stimulation with the active vitamin D metabolite 1α,25-dihydroxyvitamin D_3_ (1,25(OH)_2_D_3_). Taking all four time points together, 662 target genes were identified and segregated either by time of differential gene expression into 179 primary and 483 secondary targets or by driver of expression change into 293 direct and 369 indirect targets. The latter classification revealed that more than 50% of target genes were primarily driven by the cells' response to ex vivo exposure than by the nuclear hormone and largely explained its down-regulatory effect. Functional analysis indicated vitamin D’s role in the suppression of the inflammatory and adaptive immune response by down-regulating ten major histocompatibility complex class II genes, five alarmins of the S100 calcium binding protein A family and by affecting six chemokines of the C-X-C motif ligand family. Taken together, studying time-resolved responses allows to better contextualize the effects of vitamin D on the immune system.

## 1. Introduction

Vitamin D_3_ is a micronutrient that is either produced endogenously in UV-B exposed skin [1] or taken up by diet as well as direct supplementation [2]. The evolutionarily oldest role of vitamin D is to maintain energetic and survival homeostasis [3], while its physiologically best known function is the homeostasis of calcium levels being critical for bone mineralization [4]. In addition, another function of vitamin D is the modulation of the immune system [5]. This is important for an efficient response of innate immunity to infectious diseases, such as tuberculosis [6] or COVID-19 (coronavirus disease) [7], as well as for the avoidance of overreactions of adaptive immunity, such as in the onset and progression of the autoimmune disease multiple sclerosis [8,9] or in the context of severe forms of COVID-19 [10].

The vitamin D_3_ metabolite 1,25(OH)_2_D_3_ binds and activates the transcription factor VDR (vitamin D receptor) [11,12], i.e., vitamin D has direct effects on gene regulation [13]. VDR belongs the nuclear receptor superfamily [14] and has up to a few hundred specific target genes in about half of human tissues and cell types [15,16]. The biological function of vitamin D_3_ in health and disease is therefore directly linked to 1,25(OH)_2_D_3_-dependent changes of the transcriptome in VDR expressing cells [15]. The vitamin D-triggered transcriptome has been studied in a number of in vitro cell culture models [17,18,19,20], such as in THP-1 monocytic leukemia cells [21]. Since primary cells are far closer to the human in vivo situation, PBMCs are an attractive alternative to THP-1 cells and accessible with minimal harm to the donor [22]. PBMCs are composed of monocytes, natural killer cells, T and B cells, i.e., of innate and adaptive immune cells, of which monocytes are the most vitamin D responsive cell type [23].

The genome-wide binding pattern of VDR is known for a number of human in vitro cell culture systems representing macrophage-like cells [24], monocytes [17,25], lymphocytes [26], colorectal cancer cells [27], hepatic stellate cells [28] and prostate cells [29]. In monocytes the VDR cistrome has more than 10,000 loci, but only a few hundred persistent VDR binding sites are always occupied [25]. The latter sites are the genome’s primary contact points with 1,25(OH)_2_D_3_ and coordinate its spatio-temporal response to the nuclear hormone. The chromatin model of vitamin D signaling [30,31] had been proposed based on data obtained primarily in THP-1 cells being stimulated for 24 h with 1,25(OH)_2_D_3_. The model suggests that a primary vitamin D target gene is modulated in its expression, when the topologically associated domain (TAD), in which the gene is located, carries a prominent VDR binding site. Since TADs have a size between 100 kb and 2 Mb, this determines the maximal distance between an enhancer containing a VDR binding site and the transcription start site of the respective vitamin D target gene.

This study describes changes of the transcriptome of freshly isolated human PBMCs that had been stimulated for 4, 8, 24 and 48 h with 1,25(OH)_2_D_3_. The analysis of time-resolved transcriptional responses allows a wider view on the effects of vitamin D on these immune cells, such as a segregation into primary and secondary target genes as well as direct and indirect targets, in the context of inflammatory responses of the innate immune system and its crosstalk with the adaptive immune system.

## 2. Results

### 2.1. Time-Resolved Transcriptome Changes of PBMCs in Response to 1,25(OH)_2_D_3_

**Figure 1:**
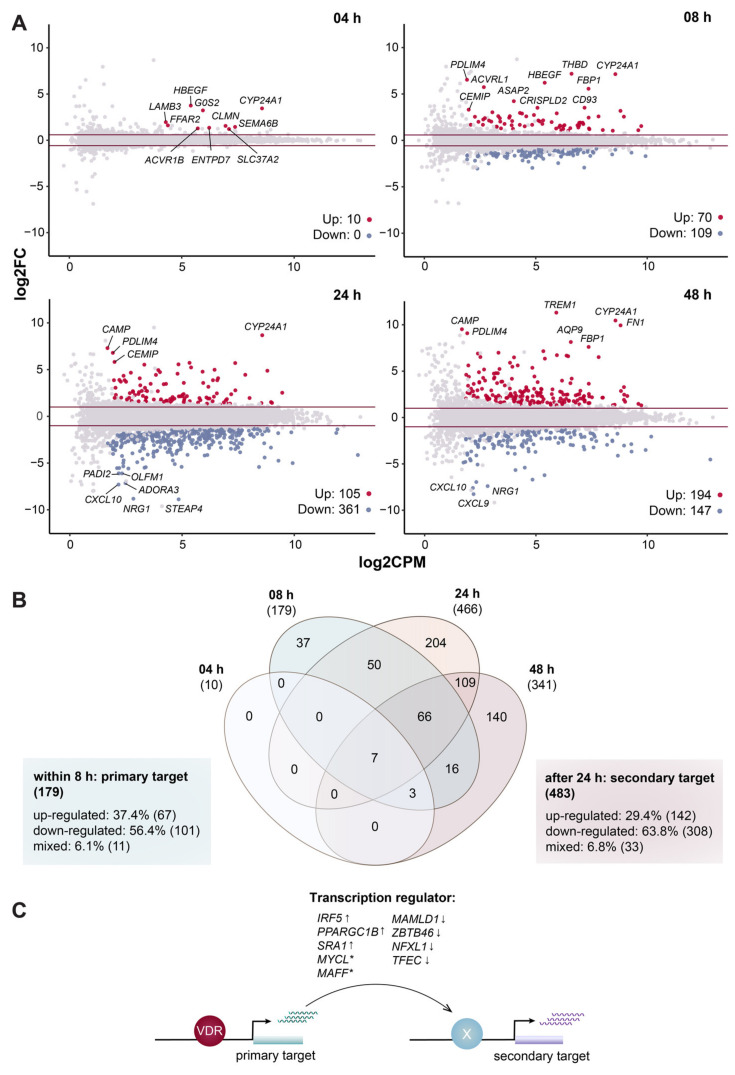
Differential gene expression. Mean-Difference (MA) plots monitor the effects of treatment (10 nM 1,25(OH)_2_D_3_) at indicated time points in comparison to solvent (0.1% EtOH) (**A**). The statistical test for differential expression was conducted as a pairwise comparison for each time point by using *EdgeR’s glmTreat* method. The difference in expression change (log2FC) for each tested gene was compared with the mean expression level between the compared groups (log2CPM). Significantly (FDR < 0.05) up- and down-regulated genes are highlighted in red and blue, respectively. At each time point the top 10 genes with highest absolute log2FC are labeled. Horizontal lines (red) indicate the applied statistical testing thresholds (absolute FC > 1.5 at 4 and 8 h and absolute FC > 2 at 24 and 48 h). A Venn diagram displays the in total 662 genes identified at different time points (**B**). Genes responding already at the early time points (4 and 8 h) are considered as primary targets. The direction of regulation of a gene over all time points was determined by applying a FC threshold of 1.5. The classic model of vitamin D signaling suggests that primary target genes encoding for the indicated transcriptional regulators mediate the activation or repression of secondary targets (**C**). Asterisks (*) denote genes with mixed FC profiles. Arrows indicate transcription start sites.

PBMCs from an healthy individual were treated immediately after isolation for 4, 8, 24 and 48 h with either 10 nM 1,25(OH)_2_D_3_ or solvent (0.1% ethanol (EtOH)). Total RNA was extracted and RNA-sequencing (RNA-seq) applied. Classical pairwise comparison of time-matched treated versus control cells detected 10 and 179 differentially expressed genes at the early time points 4 and 8 h (fold change (FC) > 1.5, false discovery rate (FDR) < 0.05, glmTreat test) as well as 466 and 341 genes at time points 24 and 48 h (FC > 2, FDR < 0.05) (Figure 1A and Table S1). The 466 target genes found after 24 h stimulation with 1,25(OH)_2_D_3_ showed more than 85% overlap with the 676 and 625 vitamin D target genes identified in PBMCs from one [32] or five individuals [33] treated under the same protocol and the 1203 genes highlighted in THP-1 cells [19] (Figure S1).

In total, of all four time points, 662 genes were differentially expressed (Table S1). However, only the genes *CYP24A1* (cytochrome P450 family 24 subfamily A member 1) (Figure S2A), *ACVR1B* (activin A receptor type 1B), *CLMN* (calmin), *ENTPD7* (ectonucleoside triphosphate diphosphohydrolase 7), *G0S2* (G0/G1 switch 2), *HBEGF* (heparin binding EGF like growth factor) and *SEMA6B* (semaphorin 6B) passed the statistical testing threshold (FC > 1.5 and > 2 at FDR < 0.05) at all four time points (Figure 1B). The genes *FFAR2* (free fatty acid receptor 2)*, LAMB3* (laminin subunit beta 3) and *SLC37A2* (solute carrier family 37 member 2) were also identified as very early targets of vitamin D, but missed the statistical threshold at time point 24 h. A similar discontinuous pattern was observed for *CD14* (CD14 molecule) and 13 other genes, which were significantly differentially expressed at 8 and 48 h but their absolute FC dropped below 1.5 at 24 h (Figure S3E,F). By contrast, 66 genes responded at the consecutive time points 8, 24 and 48 h, 50 genes at 8 and 24 h and 109 genes at 24 and 48 h. This implies that the majority (411) of the 662 differentially expressed genes were considered vitamin D targets based only on one time point, at which statistically significant response to 1,25(OH)_2_D_3_ had been observed.

The 179 genes that were within the first 8 h significantly regulated by 1,25(OH)_2_D_3_ were considered as primary targets, while the remaining 483 genes were classified as secondary targets (Figure 1B). The latter genes are assumed to be regulated by primary vitamin D target genes that encode transcription factors and/or chromatin modifiers [34], such as the transcription factors IRF5 (interferon regulatory factor 5), MAFF (MAF BZIP transcription factor F), MYCL (MYCL proto-oncogene, BHLH transcription factor), NFXL1 (nuclear transcription factor, X-box binding like 1) and TFEC (transcription factor EC) as well as the transcriptional co-regulators MAMLD1 (mastermind like domain containing 1), PPARGC1B (PPARG coactivator 1 beta), SRA1 (steroid receptor RNA activator 1) and ZBTB46 (zinc finger and BTB domain containing 46) (Figure 1C). Moreover, 209 genes were up-regulated by 1,25(OH)_2_D_3_ (58 persistent primary (Figure S2A), 130 persistent secondary (Figure S2C), two transient primary (Figure S3A), four persistent secondary (Figure S3B), seven discontinuous primary (Figure S3E) and eight discontinuous secondary (Figure S3F)) and 409 were down-regulated (74 persistent primary (Figure S2C), 208 persistent secondary (Figure S2D), 27 transient primary (Figure S3C) and 98 persistent secondary (Figure S3D) and two discontinuous secondary (Figure S3F)). In contrast, 44 genes displayed a mixed profile (11 primary (Figure S3G) and 33 secondary (Figure S3H)), i.e., they appeared to be up- or down-regulated depending on the time point. Interestingly, at the first (4 h) and last (48 h) time point 100 and 56.9% of the genes were up-regulated, while 60.9 and 77.5% of the genes were down-regulated at time points 8 and 24 h.

In summary, classical differential expression analysis over four time points identified 179 primary targets of 1,25(OH)_2_D_3_ and 483 secondary targets in PBMCs. The majority of these genes are down-regulated.

### 2.2. Distinction between Stimulating and Stabilizing Effects of 1,25(OH)_2_D_3_

**Figure 2:**
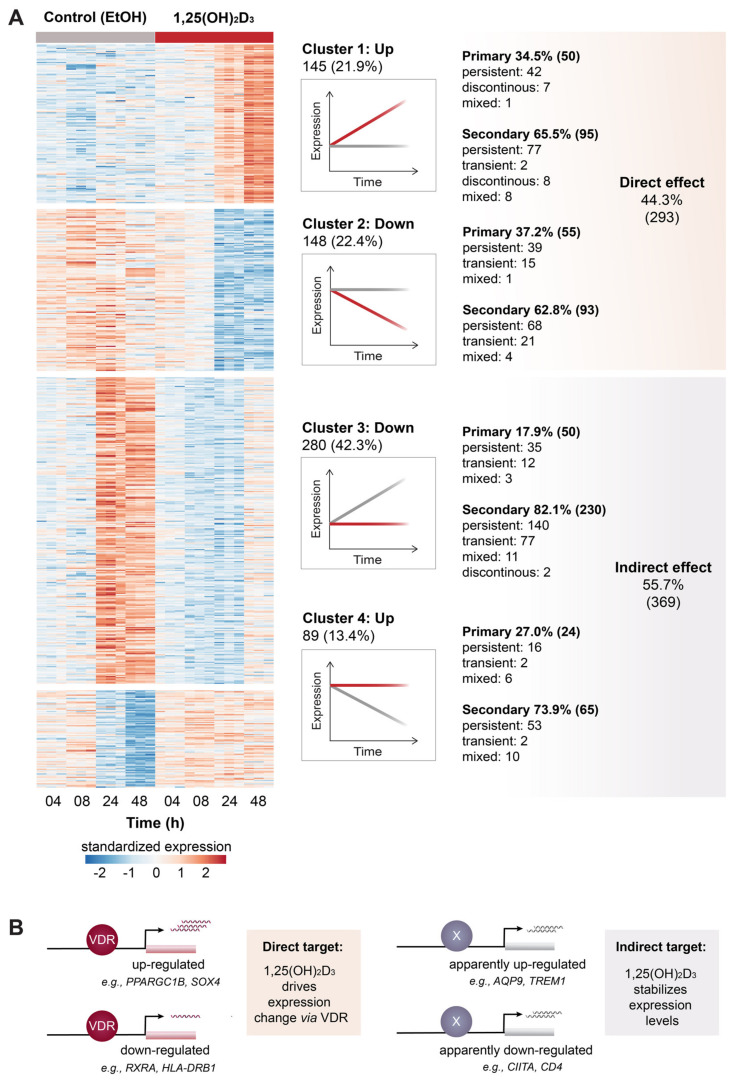
Clustering of vitamin D target genes. The machine learning algorithm *k*-means revealed four distinct clusters of vitamin D target genes based on cause and direction of expression change (**A**). 1,25(OH)_2_D_3_-driven expression changes indicated direct targets (clusters 1 and 2), whereas genes whose expression was primarily stabilized in the presence of 1,25(OH)_2_D_3_ are indirect targets (clusters 3 and 4). This suggests an alternative view on vitamin D signaling: 1,25(OH)_2_D_3_ either directly induces or reduces via VDR the expression of its target genes (**B**, **left**) or prevents their expression change mediated by other factors (**B**, **right**).

A focus on the differences in mRNA levels of treated and control samples is important for the detection of statistically significant effects, but it hides the underlying gene expression patterns. Therefore, the unsupervised machine learning technique *k*-means was applied, which segregated the expression pattern information of all 662 vitamin D target genes into four clusters (Figure 2A). Cluster 1 comprises 145 directly up-regulated genes (examples in Figure S4A) and cluster 2 contains 148 directly down-regulated genes (examples in Figure S4B). In contrast, the 280 and 89 genes of clusters 3 and 4 are stabilized in their expression by 1,25(OH)_2_D_3_, while without treatment the genes are up- or down-regulated, respectively (examples in Figure S4C,D). Displaying the expression trajectories provides a better overview on the responses of the in total 131 genes with a transient FC profile (Figure S5A–D), of 17 genes with a discontinuous FC profile (Figure S5E,F) and of 44 genes with a mixed FC profile (Figure S5G,H). This indirect effect of 1,25(OH)_2_D_3_ signaling applies for more than half (55.7%) of differentially expressed genes. In particular, down-regulated genes are 3.1-times more likely indirect vitamin D targets than up-regulated genes (FDR < 0.001, Fisher's exact test (FET)). By contrast, primary vitamin D responding genes are 2.2-times more often found to be direct targets (FDR < 0.001, FET). The latter tendency is also visible in multidimensional scaling (MDS) plot (Figure S6), which displays within the first 8 h a larger divergence between 1,25(OH)_2_D_3_-treated samples than control samples, i.e., vitamin D-driven changes precede cell culture-related effects. Thus, direct effects of 1,25(OH)_2_D_3_ are very likely mediated by VDR binding to the regulatory regions of the respective direct vitamin D target genes, while indirect target genes may be the consequence of more global effects, such as changes in the epigenetic landscape (Figure 2B).

Taken together, the analysis PBMC gene expression after treatment with 1,25(OH)_2_D_3_ and solvent using clustering analysis allows a dissection of the vitamin D responding genes into 293 direct and 369 indirect targets.

### 2.3. Functional Impact of Vitamin D Target Genes

**Figure 3:**
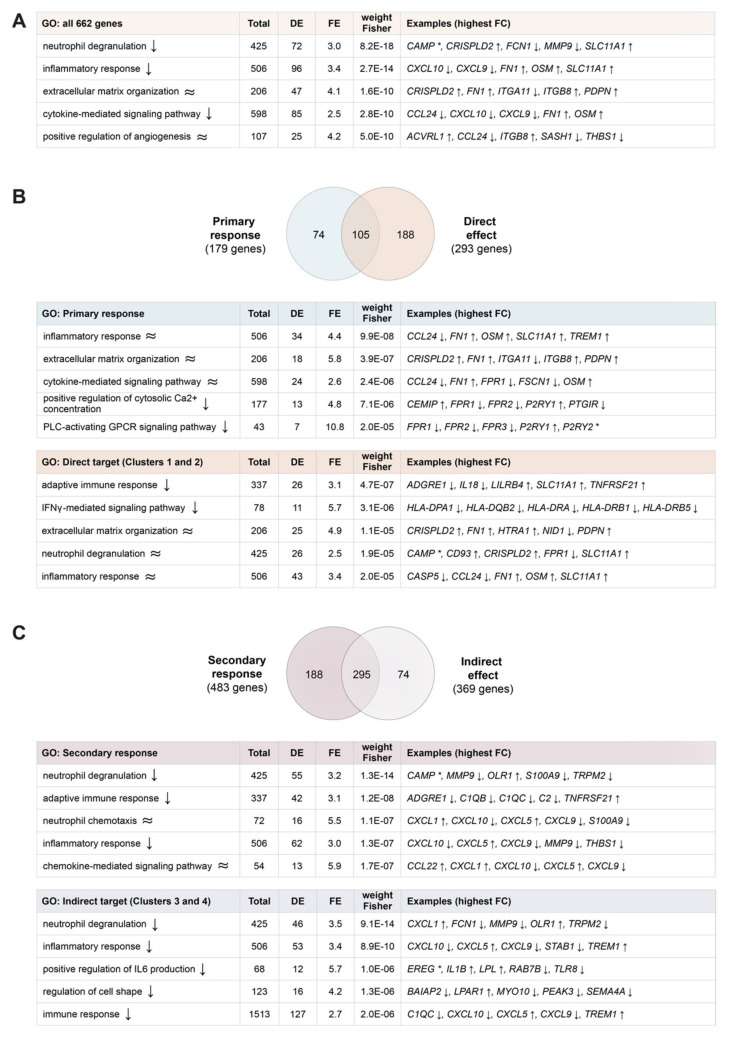
Biological processes affected by vitamin D. GO term enrichment was performed for all 662 vitamin D target genes (**A**), primary and direct targets (**B**) as well as for secondary and indirect targets (**C**). The list of all 12,305 expressed genes served as reference. Venn diagrams indicate the overlap of primary and direct targets (**B**) as well as secondary and indirect targets (**C**). The top 5 enriched terms (ranked by statistical significance) and the corresponding top 5 representative genes (based on absolute median FC) are indicated. The direction of regulation of a gene is based on the FC across all four time points. Asterisks (*) denote genes with mixed FC profiles. Arrows indicate direction of regulation of pathways and target genes.

The functional impact of the changes in the expression of the identified 662 vitamin D target genes was analyzed via Gene Ontology (GO) term enrichment (Figure 3A). Accordingly, 1,25(OH)_2_D_3_ down-regulates “neutrophil degranulation”, “inflammatory response” as well as “cytokine-mediated signaling pathway” and affects “extracellular matrix organization” as well as “positive regulation of angiogenesis”. The 179 primary vitamin D target genes influence “inflammatory response”, “extracellular matrix organization” as well as “cytokine-mediated signaling pathway” and down-regulate “positive regulation of cytosolic Ca^2+^ concentration” as well as “PLC (phospholipase C)-activating GPCR (G protein-coupled receptor) signaling pathway” (Figure 3B). Since 105 primary target genes are also direct vitamin D targets, both set of genes modulate “extracellular matrix organization” and “inflammatory response”. In addition, direct vitamin D target genes down-regulate “adaptive immune response” and “IFNγ (interferon gamma)-mediated signaling pathway” and affect “neutrophil degranulation”. The 483 secondary vitamin D target genes down-regulate “neutrophil degranulation”, “adaptive immune response” as well as “inflammatory response” and affect “neutrophil chemotaxis” as well as “chemokine-mediated signaling pathway” (Figure 3C). Of this gene set 295 members are also indirect vitamin D targets and both groups of genes agree on the down-regulation of “neutrophil degranulation” and “inflammatory response”. Furthermore, indirect target genes down-regulate “positive regulation of IL6 (interleukin 6) production”, “regulation of cell shape” and “immune response”. In a more focused approach, the 17 different genes representing the top five genes mediating the top five functions of primary target genes were compared with representative 21 direct target genes (Figure S7, left). The most representative unique function of the primary target genes is “PLC-activating GPCR signaling pathway” and “IFNγ-mediated signaling pathway” for direct target genes, while “inflammatory response” is a common function. In the same way the 16 most representative secondary target genes were compared with 21 indirect target genes (Figure S7, right), which resulted in the pathways “chemokine-mediated signaling pathway” and “positive regulation of IL6 production” representing uniquely secondary targets and indirect targets, respectively, while “neutrophil degranulation” being common for both.

In contrast, the segregation of the proteins encoded by all target genes (Figure S8A), primary target genes (Figure S8B), secondary target genes (Figure S8C), direct target genes (Figure S8D) and indirect target genes (Figure S8E) into functional categories, such as enzymes, structural proteins, transcriptional regulators, signal transduction regulators, secreted factors or membrane receptors, did not indicate any significant differences between the five different gene sets.

In summary, GO term enrichment analysis indicated that vitamin D majorly down-regulates “inflammatory response”, “neutrophil degranulation” and “adaptive immune response”.

### 2.4. Colocalization of Vitamin D Target Genes with VDR Binding Sites

**Figure 4:**
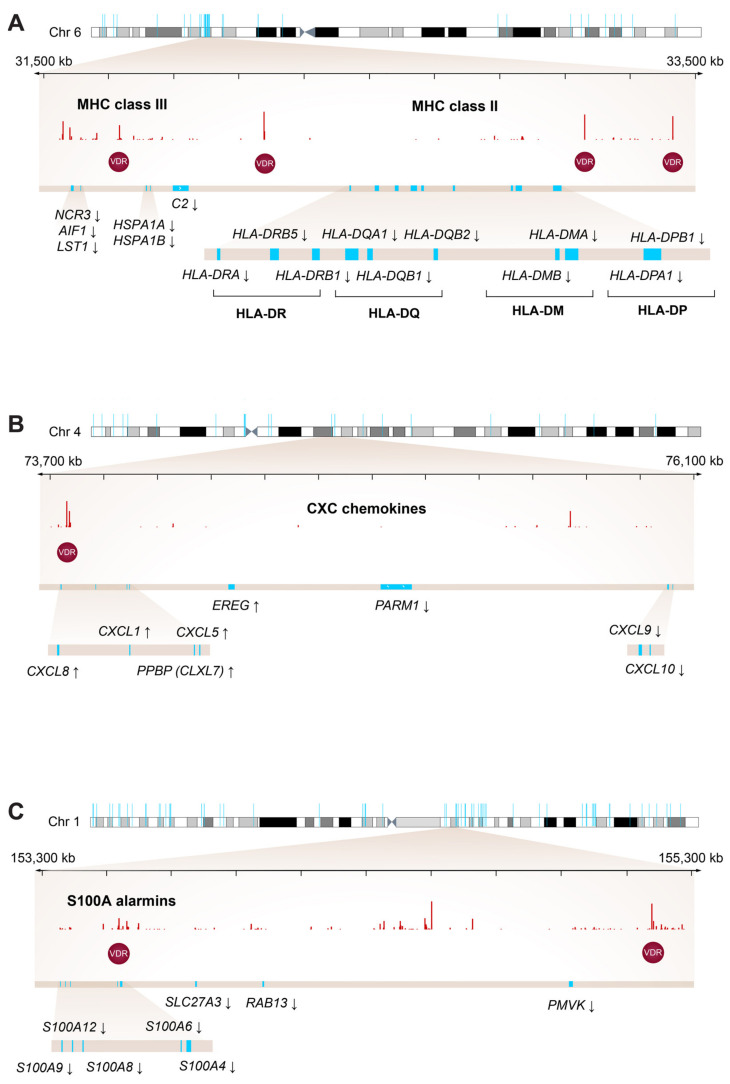
Hotspots of vitamin D signaling. Karyoplots showing the location of vitamin D target genes (blue) highlight regions encoding for *HLA* genes in chromosome 6 (**A**), *CXCL* genes in chromosome 4 (**B**) and *S100A* genes in chromosome 1 (**C**). VDR ChIP-seq data from THP-1 cells [25] monitor 1,25(OH)_2_D_3_-triggered VDR binding at indicated regions. Persistent binding sites of VDR are highlighted by red circles. Arrows indicate direction of regulation of target genes.

The inspection of the lists of vitamin D target genes representing the major biological processes regulated by 1,25(OH)_2_D_3_ in PBMCs (Figure 3 and Figure S7) highlighted a couple of gene families with key impact on the function of the immune system. Importantly, 10 of 12 genes that encode for the α- and β-chains of the major histocompatibility complex (MHC, also named human leukocyte antigen (HLA)) class II proteins, *HLA-DRA*, *HLA-DRB1*, *HLA-DRB5*, *HLA-DQA1*, *HLA-DQB1*, *HLA-DQB2*, *HLA-DMA*, *HLA-DMB*, *HLA-DPA1* and *HLA-DPB1*, are persistently down-regulated vitamin D target genes (Figure 4A). Interestingly, with the exception of *HLA-DMA* and *HLA-DMB* all are also direct targets. The *HLA* genes are located in a dense cluster with the down-regulated vitamin D target genes *NCR3* (natural cytotoxicity triggering receptor 3), *AIF1* (allograft inflammatory factor 1), *LST1* (leukocyte specific transcript 1), *HSPA1A* (heat shock protein family A (Hsp70) member 1A), *HSPA1B* and *C2* (complement C2). Within this genomic region of approximately 2 Mb in chromosome 6, four persistent VDR binding sites are known [25]. Furthermore, from the family of C-X-C motif chemokine ligands (CXCLs) in chromosome 4, a similarly sized genomic region contains the up-regulated vitamin D target genes *CXCL1*, *CXCL5*, *CXCL7* (official gene name *PPBP* (pro-platelet basic protein)), *CXCL8* and *EREG* (epiregulin) as well as the down-regulated genes *CXCL9*, *CXCL10* and *PARM1* (prostate androgen-regulated mucin-like protein 1) (Figure 4B). In this region there is only one persistent VDR binding site. Moreover, from the *S100A* (S100 calcium binding protein A) genes of the alarmin family in chromosome 2 *S100A4*, *S100A6*, *S100A8*, *S100A9* and *S100A12* as well as the genes *SLC27A3* (solute carrier family 27 member 3), *RAB13* (RAB13, member RAS oncogene family) and *PMVK* (phosphomevalonate kinase) are down-regulated by 1,25(OH)_2_D_3_ (Figure 4C). In this 2 Mb region two persistent VDR binding sites are found.

Taken together, the functional impact of 1,25(OH)_2_D_3_ stimulation of PBMCs is prominently mediated via the regulation of clusters of *HLA*, *CXCL* and *S100A* gene families.

## 3. Discussion

This study describes the temporal changes of the transcriptome of human PBMCs in response to 1,25(OH)_2_D_3_ analyzed by classical differential gene expression as well as via clustering analysis. Compared to traditional analysis of a single treatment time, such as 24 h [33,35], the use of multiple time points does not only identify a higher number of vitamin D target genes, but also allows differentiating them either into 179 primary and 483 secondary targets or into 293 direct and 369 indirect targets. Although there is reasonable overlap between primary and direct vitamin D target genes as well as between secondary and indirect targets, the terms represent different mechanistic conceptions of vitamin D signaling. The essential condition of a primary vitamin D target gene is that within the same TAD an enhancer with a VDR binding site is found [34]. In contrast, a secondary vitamin D target gene does not require the presence of a VDR bearing enhancer, but its transcriptional activity is controlled by transcription factors, co-factors or chromatin modifiers that are encoded by primary vitamin D target genes. For comparison, a direct vitamin D target gene is either up- or down-regulated by 1,25(OH)_2_D_3_ in relation to a time-matched solvent control, i.e., the VDR ligand affects gene expression over time, while the control sample does not change. The opposite applies for indirect target genes, which are primarily responding to environmental conditions, such as cellular stress due to isolation and ex vivo culture of PBMCs but not to 1,25(OH)_2_D_3_. In this context, 1,25(OH)_2_D_3_ has a stabilizing effect on the transcriptome, i.e., vitamin D counteracts transcriptional changes triggered by other factors. Thus, more than half of the 662 target genes are responding indirectly to vitamin D treatment.

Persistently up-regulated primary vitamin D target genes are mechanistically well understood via VDR actively changing the epigenetic landscape and regulating gene transcription as described in the chromatin model of vitamin D signaling [16,31]. This study demonstrates that in human PBMCs the model applies only to 8.8% of the genes responding to 1,25(OH)_2_D_3_. Nevertheless, the list of these 58 persistently up-regulated primary vitamin D targets contains a number of well-known genes, such as *CYP24A1* and *FBP1* (fructose bisphosphatase 1), which have been reported in numerous studies [36,37]. In contrast, there is no general model explaining the down-regulation of genes by vitamin D. Every gene has an individual scenario for its up-regulation by a set of transcription factors and chromatin modifiers, which can be counteracted in many ways by VDR and its ligand. The majority of down-regulated target genes are classified as indirect targets, i.e., 1,25(OH)_2_D_3_ rather counteracts their up-regulation than prominently down-regulates their expression. Since most of the 662 vitamin D responding genes are indirect targets, this also implies that their regulation is context dependent, such as the health and lifestyle situation of the individual. Moreover, 192 genes show unusual transcriptional dynamics, such as transient, discontinuous or mixed FC profiles. Most these genes are indirect vitamin D targets, i.e., their unusual behavior is primarily due to changes of the reference samples. Thus, the analysis of gene expression over time allows a far more differential description and understanding of vitamin D responding genes than snapshots of vitamin D effects at a single time point.

Functional analysis of all 662 targets identified in this study in comparison to the subgroups of primary, secondary, direct and indirect genes suggests that the overall effect of vitamin D treatment is the down-regulation of inflammatory response, neutrophil degranulation and adaptive immune response. Since neutrophil degranulation involves the release of anti-microbial and inflammatory proteins [38], this process rather represents an additional aspect of inflammatory response than a separate function. For example, the vitamin D target genes *CXCL1*, *CXCL5*, *CXCL8* and *PPBP* (*CXCL7*) encode for CXCL chemokine family members that act as neutrophil chemoattractants [39]. Since inflammation-related processes are predominantly mediated by secondary and indirect target genes, they seem to represent a context-specific effect of vitamin D. In parallel, vitamin D suppresses the production of other inflammatory molecules that are triggered by acute infections or inflammatory disorders. For example, vitamin D could reduce ARDS (acute respiratory distress syndrome) in the context of COVID-19 by suppressing the expression of target genes, such as *IL18*, *CXCL9* and *CXCL10*, that mediate the cytokine storm associated with the severe form of the disease [40,41]. This is also in line with the down-regulation of damage-associated molecular patterns (also called alarmins) of the S100A family, such as those encoded by the direct vitamin D target genes *S100A8*, *S100A9* and *S100A12*, that are highly expressed and released by neutrophils and inflammatory monocytes/macrophages during tissue damage or cellular stress reactions [42,43]. The genes *S100A8* and *S100A9* are prominently up-regulated in inflammatory diseases being associated with phagocyte activation, like autoimmune and autoinflammatory disorders, infections, allergies, cardiovascular diseases and cancer [43,44]. Given their involvement in disease pathology, the inhibition of *S100A8* and *S100A9* gene expression may be important for the treatment of various inflammation-associated disorders. Interestingly, S100A8, S100A9 and S100A12 protein levels are used as sensitive biomarkers for monitoring disease activity in juvenile idiopathic arthritis, inflammatory bowel disease and psoriasis.

The regulation of adaptive immune response is another important function of vitamin D in PBMCs. It is mediated by later responding but direct target genes, many of which many are also associated with IFNγ-mediated signaling. Importantly, this study demonstrated that 10 of 12 *HLA* genes encoding for MHC-II proteins are direct down-regulated vitamin D targets. MHC-II proteins are expressed on the surface of antigen-presenting cells, such as dendritic cells, monocytes/macrophages and B cells, through which they present CD4^+^ T helper (T_H_) cells peptide antigens [45]. This is further enhanced by the down-regulation of the genes *ICAM1* (intercellular adhesion molecule 1), *CIITA* (class II major histocompatibility complex transactivator) as well as *CD4*, which encode for a co-stimulator of T cell activation, the master regulator of *HLA* gene transcription and the main co-receptor of the T cell receptor, respectively [46]. Thus, vitamin D reduces the functionality of the immunological synapse between antigen-presenting cells and T_H_ cells and lowers the risk of overboarding adaptive immune responses in this way. This may provide a mechanistic explanation of vitamin D's beneficial role in the context of autoimmune diseases [47]. Interestingly, the most prominently down-regulated vitamin D target gene of the *HLA* family, *HLA-DRB1,* is known as the major risk gene for multiple sclerosis. Since the risk variant HLA-DRB1*15:01 leads to high HLA-DRB1 protein levels, its down-regulation by vitamin D may serve as a therapeutic target [48,49]. Notably, vitamin D-mediated down-regulation of *HLA-DRB1* has been observed in PBMCs from different individuals [33] and thus may not be restricted to certain genotypes as suggested earlier [49].

Persistent VDR binding sites are reasonably conserved between comparable cellular models, such as PBMCs and THP-1 cells [24,25]. Therefore, they offer the core of a mechanistic understanding of the regulation of primary as well as of direct vitamin D target genes. In this study, this was exemplified for the clusters of *HLA*, *CXCL* and *S100A* genes, which have four, one and two persistent VDR binding sites in their neighborhood, respectively. Thus, it can be assumed that ligand-activated VDR modulates the epigenetic landscape at the respective genomic regions and explains the response of the gene clusters to vitamin D.

The focus of this study was on the effects of vitamin D stimulation of PBMCs immediately after their isolation. Since cell sorting induces stress to cells [50], the RNA-seq approach of this study captured only the bulk response of PBMCs, i.e., the heterogeneity of the cell population is masked. Thus, the most elegant approach would have been transcriptome profiling via single cell RNA-seq. However, the majority of the vitamin D target genes are primarily expressed in monocytes, i.e., the results presented in this study primarily reflect that of the monocyte fraction of PBMCs. A further limitation of this study is its focus on the transcriptome, which is only a limited proxy for protein levels [51]. Thus, the findings of this study and their functional implications need to be confirmed with proteome-wide data and functional essays. Finally, while substantial differential expression only occurred after 8 h, a denser sampling at earlier time points would allow a more precise dissection between direct and indirect targets. This applies in particular to very early responding vitamin D target genes, such as *G0S2,* which display significant differential expression already after 4 h but their expression levels are not changing at later time points. Thus, some very early responding genes may have been mislabeled as indirect targets.

In conclusion, this study describes the time-resolved transcriptional response of human PBMCs to vitamin D stimulation. This experimental design allows the segregation of the 662 vitamin D responding genes into primary, secondary, direct and indirect target genes. Prominent vitamin D targets are the clusters of the *HLA*, *CXCL* and *S100A* genes, which mediate inflammatory processes of the innate immune system as well as the response of the adaptive immune system.

## 4. Materials and Methods

### 4.1. Sample Collection

Peripheral blood was collected from a single healthy individual (male, age 56 years) that participated in the VitDHiD trial (NCT03537027) [52].

### 4.2. PBMC Isolation and Stimulation

PBMCs were isolated immediately after collecting 20 mL peripheral blood using Vacutainer CPT Cell Preparation Tubes with sodium citrate (Becton Dickinson) according to manufacturer’s instructions. After washing with phosphate-buffered saline the cells were grown at a density of 0.5 million/mL in 5 mL RPMI 1640 medium supplemented with 10% charcoal-depleted fetal calf serum, 2 mM L-glutamine, 0.1 mg/mL streptomycin and 100 U/mL penicillin at 37 °C in a humidified 95% air/5% CO_2_ incubator and treated for 4, 8, 24 or 48 h with either 10 nM 1,25(OH)_2_D_3_ (Sigma-Aldrich) or solvent (0.1% EtOH). All experiments were performed in three repeats. Deconvolution of RNA-seq data using the algorithm CIBERSORTx [53] and its LM6 signature matrix estimated the relative amounts of B cells (7%), CD8^+^ T cells (32%), CD4^+^ T cells (20%), natural killer cells (21%) and monocytes/macrophages (20%) within the PBMC pool.

### 4.3. RNA-Seq Data Generation and Processing

Total RNA was isolated using the High Pure RNA Isolation Kit (Roche) according to manufacturer’s instructions. RNA quality was assessed on an Agilent 2100 Bioanalyzer system (RNA integrity number ≥ 8). rRNA depletion and cDNA library preparation were performed using the New England Biolabs kits NEBNext rRNA Depletion, NEBNext Ultra II Directional RNA Library Prep for Illumina and NEBNext Multiplex Oligos for Illumina (Index Primers Sets 1 and 2) according to manufacturer’s protocols. RNA-seq libraries went through quality control on an Agilent 2100 Bioanalyzer and were sequenced on a NextSeq 500 system (Illumina) at 75 bp read length using standard protocols at the Gene Core facility of the EMBL (Heidelberg, Germany). All samples were prepared and sequenced as one batch.

Single-end, reverse-stranded cDNA sequence reads were aligned to the reference genome (version GRCh38) with Ensembl annotation (version 103) by using default settings of the nf-core/rnaseq STAR-Salmon pipeline (version 3.0) (http://doi.org/10.5281/zenodo.4323183, accessed on 25 June 2021) [54]. The number of nucleotide sequence reads are shown in Table S2. Ensembl gene identifiers were annotated with gene symbol, description, genomic location and biotype by accessing the Ensembl database (version 104) via the R package *BiomaRt* (version 2.46.0) [55]. Ensembl gene identifiers missing HGNC gene symbol annotation, Entrez ID, genomic location information or being mitochondrially encoded were removed from the datasets. When a gene name appeared more than once, the entry with the highest average gene counts was kept.

### 4.4. Transcriptome Data Analysis

Differential gene expression analysis was computed in R (version 4.0.5) in the CentOS 7 Linux operating system using the tool *EdgeR* (version 3.32.1) [56]. The analysis focused on the 19,147 protein-coding genes, in order to mitigate transcriptional noise expected by non-coding genes. Read counts were normalized for differences in library size to counts per million (CPM). Genes with very low expression were filtered out by applying the function *FilterByExpr()*, in order to mitigate the multiple testing problem and to not interfere with the statistical approximations of the *EdgeR* pipeline. This requirement was fulfilled by 12,305 genes. After filtering, library sizes were recomputed and trimmed mean of M-value normalization was applied. The transcriptome data structure was explored via the dimensionality reduction method MDS (Figure S6). MDS was computed via *EdgeR*’s function *plotMDS()*, in which distances approximate the typical log2 fold change (FC) between the samples. This distance was calculated as the root mean square deviation (Euclidean distance) of the largest 500 log2FCs between a given pair of samples, i.e., for each pair a different set of top genes was selected. The inspection of the MDS plot showed a time-dependent divergence from the native transcriptome state and its modulation by 1,25(OH)_2_D_3_ treatment as the principal two factors driving differences in PBMCs gene expression profiles. The gene-wise statistical test for differential expression was computed using the generalized linear model quasi-likelihood pipeline [57]. Trended negative binomial dispersion estimate was calculated using the method CoxReid profile-adjusted likelihood and together with empirical Bayes-moderated quasi-likelihood gene-wise dispersion estimates used for generalized linear model fitting. The empirical Bayes shrinkage was robustified against outlier dispersions as recommended [57]. The *glmTreat* approach was used to test for differential expression relative to FC > 1.5 at the early time points 4 and 8 h and FC > 2 at 24 and 48 h. A classical pairwise comparison was chosen for its superior performance in time course RNA-seq data analysis [58]. Genes with a Benjamini–Hochberg corrected *p*-value, i.e., false discovery rate (FDR) < 0.05 and a total trimmed mean of M value-normalized CPM count > 25 (i.e., sum of the average gene expression level at each time point) were considered as significant vitamin D target genes (Table S1). MA plots were generated with *vizzy* (version 1.0.0, https://github.com/ATpoint/vizzy, accessed on 23 December 2021). Gene symbols from external datasets were updated with HGNC helper (version 0.8.1).

### 4.5. Clustering and Functional Analysis

The 662 vitamin D target genes (Table S1) were clustered based on their z-score standardized, log2-transformed expression values (log2CPM) using *k*-means Hartigan–Wong method with 25 random sets and a maximum of 1000 iterations implemented in R stats (version 4.0.5, modified code from [59]) and visualized with *pheatmap* (version 1.0.12). No further clustering was applied, i.e., genes within each cluster are displayed in alphabetical order. Clusters were assessed for over-representation using a two-sided FET with a Benjamini–Hochberg corrected *p*-value applying *rstatix* (0.7.0). GO term enrichment for biological processes was performed using topGO (version 2.42.0) with the default weight01 algorithm and FET (Table S3). As reference served, all genes tested for differential expression, of which 11,241 genes could be mapped to GO terms (GO.db version 3.12.1). Enriched terms with less than five significant genes were excluded from the analysis. A biological process was considered as down-regulated when the ratio of down-regulated genes to up-regulated genes was higher than 2.

Gene names were mapped to UniProtKB identifiers via *BiomaRt*. Functional attributes of all vitamin D target genes were retrieved through programmatic access (REST API) of the UniProtKB (Uniprot Knowledge Base) website using requests (version 2.25.1) and io in Python (version 3.8.8). Only manually reviewed (Swiss-Prot) entries were used for annotation. Gene product locations and functions were preferentially assigned using UniProtKB/Swiss-Prot Keywords and complemented with GO annotation (cellular component and molecular function domains). The total list of functional annotations was manually inspected and simplified into overarching terms, by which the genes were assigned with (Table S3). Unmappable entries were annotated using information provided by the integrative database GeneCards (www.genecards.org, accessed on 23 December 2021). Custom code of the analysis can be found at https://github.com/andreahanel/2021_Timecourse, accessed on 23 December 2021.

Location of genes along chromosomes was plotted using *karyoploteR* (version 1.12.4). VDR chromatin immunoprecipitation sequencing (ChIP-seq) data [25] were converted to hg38 coordinates using the UCSC liftover chain file (hg19ToHg38.over.chain.gz) via *CrossMap* (version 0.2.6) and visualized using the IGV browser [60] together with vitamin D target genes. The latter were imported to IGV in bed file form created with *GenomicRanges* (1.38.2) and *rtracklayer* (1.46.0).

## Data Availability

Fastq files of the 24 libraries can be found at Gene Expression Omnibus (GEO, www.ncbi.nlm.nih.gov/geo, accessed on 23 December 2021) with accession number GSE189984.

## Supplementary Materials

The following supporting information can be downloaded at: https://www.mdpi.com/article/10.3390/ijms23020911/s1.
