# Supplementary material for: Time-Resolved Gene Expression Analysis Monitors the Regulation of Inflammatory Mediators and Attenuation of Adaptive Immune Response by Vitamin D
Source: Int J Mol Sci. 2022 Jan 14;23(2):911. doi: 10.3390/ijms23020911 (PMC8776203; doi:10.3390/ijms23020911)
Supplement: Supplementary file 1 [file ijms-23-00911-s001.zip › Supplements/Supplements.pdf]

SUPPLEMENTARY TABLE LEGENDS

Table S1: Vitamin D target genes in PBMCs. Differentially expressed genes were identified using the statistical test *glmTreat* with the thresholds FC > 1.5 at time points 4 and 8 h and FC > 2 at 24 and 48 h. Characterization of vitamin D target genes by time and driver of expression change as well as their functional attributes, such as cellular location and molecular function. Functional information is based on manually curated UniProtKB Keywords and GO terms retrieved from the UniprotKB/Swiss-Prot database and GeneCards (www.genecards.org). Raw RNA-seq counts and normalized gene expression with statistical metrics are found at GEO with accession number GSE189984.

Table S2: Read alignment. Numbers of uniquely aligned reads of each of the 24 samples are indicated.

Table S3: Significantly enriched biological processes. GO term enrichment was performed for all, primary, secondary, direct (clusters 1 and 2) and indirect (clusters 3 and 4) vitamin D target genes using weight01 algorithm and FET in the topGO package. For the top 5 enriched terms genes are listed and sorted by absolute median FC.

SUPPLEMENTARY FIGURE LEGENDS

Fig. S1: Overlap of vitamin D target genes. A Venn diagram displays the overlap of vitamin D target genes of this study (time point 24 h) with external datasets that follow the same study protocol in PBMCs [41,42] and THP-1 cells [19].

Fig. S2: Time course profiles of persistent vitamin D target genes. Examples of vitamin D target genes with a persistent FC profile are shown and categorized as primary up-regulated (**A**), secondary up-regulated (**B**), primary down-regulated (**C**) and secondary down-regulated (**D**). The genes *CYP24A1*, *PDLIM4* (PDZ and LIM domain 4), *AQP9* (aquaporin 9), *CXCL5*, *ITGA11* (integrin subunit alpha 11), *STEAP4* (STEAP4 metalloreductase), *OLFM1* (olfactomedin 1) and *NRG1* (neuregulin 1), were selected based on highest FC. The FC threshold of 1.5 is shaded in grey.

Fig. S3: Time course profiles of vitamin D target genes with unusual dynamics. Example genes with transient (**A**-**D**), discontinuous (**E** and **F**) or mixed (**G** and **H**) log2FC profiles are shown and categorized as primary (**A**, **C**, **E** and **G**) and secondary (**B**, **D**, **F** and **H**) targets. The genes *BCL2* (BCL2 apoptosis regulator), *VCAN* (versican), *CYP27A1*, *JDP2* (Jun dimerization protein 2), *CD14*, *TNFRSF21* (TNF receptor superfamily member 21), *MYCL* and *MNDA* (myeloid cell nuclear differentiation antigen) were chosen as representatives. The FC threshold of 1.5 is shaded in grey.

Fig. S4: Gene expression trajectories highlighted by clustering. The genes *PPARGC1B*, *SOX4* (SRY-box transcription factor 4), *RXRA* (retinoid X receptor alpha), *HLA-DRB1*, *TREM1* (triggering receptor expressed on myeloid cells 1), *AQP9*, *CIITA* and *CD4*, were selected as examples demonstrating the distinction between a direct and indirect (stabilizing) effect of 1,25(OH)$_2$D$_3$. The genes were categorized as primary up-regulated (**A**), secondary up-regulated (**B**), primary down-regulated (**C**) and secondary down-regulated (**D**). Error bars indicate standard deviation.

Fig. S5: Gene expression trajectories show unusual FC dynamics. The same set of examples as in **Fig. S3** were chosen, in order to monitor the expression profiles of genes with a transient (**A**-**D**), discontinuous (**E** and **F**) or mixed (**G** and **H**) log2FC profile. The genes were categorized into primary (**A**, **C**, **E** and **G**) and secondary (**B**, **D**, **F** and **H**) targets. Bars indicate standard deviation.

Fig. S6: Sample quality assessment *via* MDS. Dimensionality reduction was applied using MDS, in order to visualize the similarities between the 24 samples. Progressive time-dependent distancing from native gene expression state in PBMCs (dimension 1) and its modulation by 1,25(OH)$_2$D$_3$ treatment (dimension 2) is shown. Distances on the plot approximate the typical log2FC between the samples, *i.e.*, one unit represents a FC of 2.

Fig. S7: Prominent genes mediating common and uniquely highlighted biological processes. Venn diagrams indicate the overlap of the top 5 genes representing the top 5 pathways (**Fig. 3**) of primary and direct (**left**) as well as secondary and indirect (**right**) responses to vitamin D. Pathways representing unique and common functions are named and the genes *FPR2* (formyl peptide receptor 2), *CXCL9*, *CCL24* (C-C motif chemokine ligand 24), *TRPM2* (transient receptor potential cation channel subfamily M member 2), *HLA-DRB1* as well as *TLR8* (Toll like receptor 8) serve as example for each of the six categories.

Fig. S8: Functional profile of proteins encoded by vitamin D target gene sets. Pie charts indicate proportions of main classes of proteins encoded by all (**A**), primary (**B**), secondary (**C**), direct (**D**) or indirect (**E**) vitamin D target genes. Protein functions were assigned using the database UniProtKB/Swiss-Prot.

Fig. S1

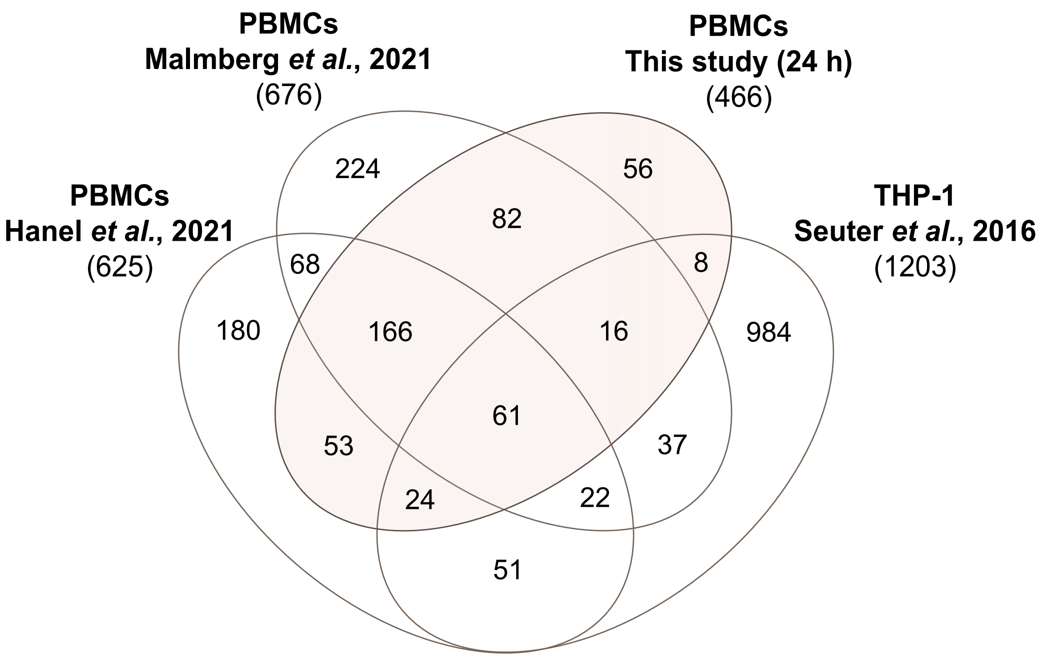
PBMCs
Malmberg *et al.*, 2021
(676)
PBMCs
This study (24 h)
(466)
PBMCs
Hanel *et al.*, 2021
(625)
THP-1
Seuter *et al.*, 2016
(1203)
224
56
82
68
8
180
166
16
984
61
53
37
24
22
51

Fig. S2

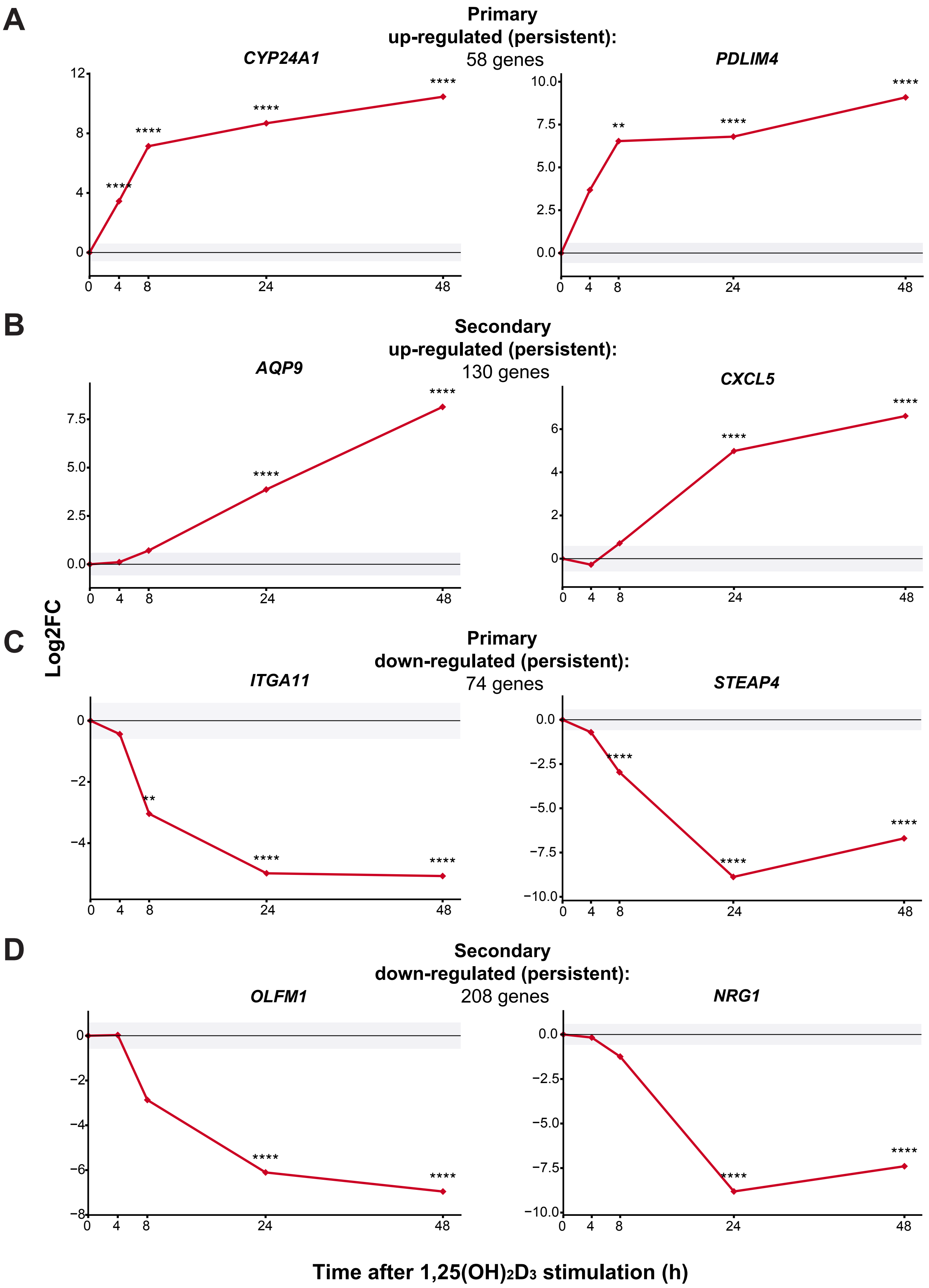
A
Primary
up-regulated (persistent):
58 genes
CYP24A1
PDLIM4
B
Secondary
up-regulated (persistent):
130 genes
AQP9
CXCL5
C
Primary
down-regulated (persistent):
74 genes
ITGA11
STEAP4
D
Secondary
down-regulated (persistent):
208 genes
OLFM1
NRG1
Log2FC
Time after 1,25(OH)2D3 stimulation (h)

Fig. S3

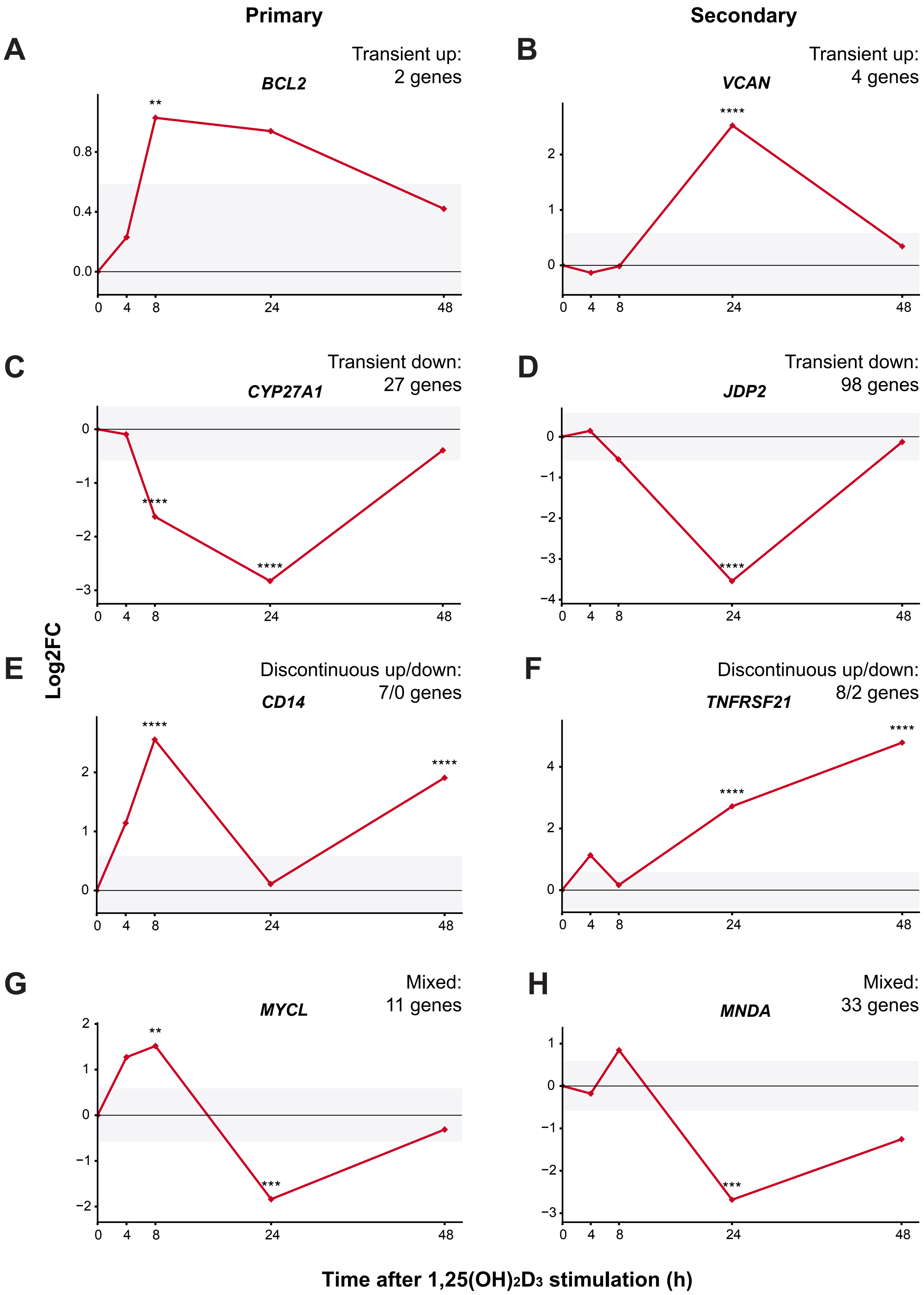
Primary
Secondary
A
Transient up:
2 genes
BCL2
B
Transient up:
4 genes
VCAN
C
Transient down:
27 genes
CYP27A1
D
Transient down:
98 genes
JDP2
E
Discontinuous up/down:
7/0 genes
CD14
F
Discontinuous up/down:
8/2 genes
TNFRSF21
G
Mixed:
11 genes
MYCL
H
Mixed:
33 genes
MNDA
Log2FC
Time after 1,25(OH)2D3 stimulation (h)

Fig. S4

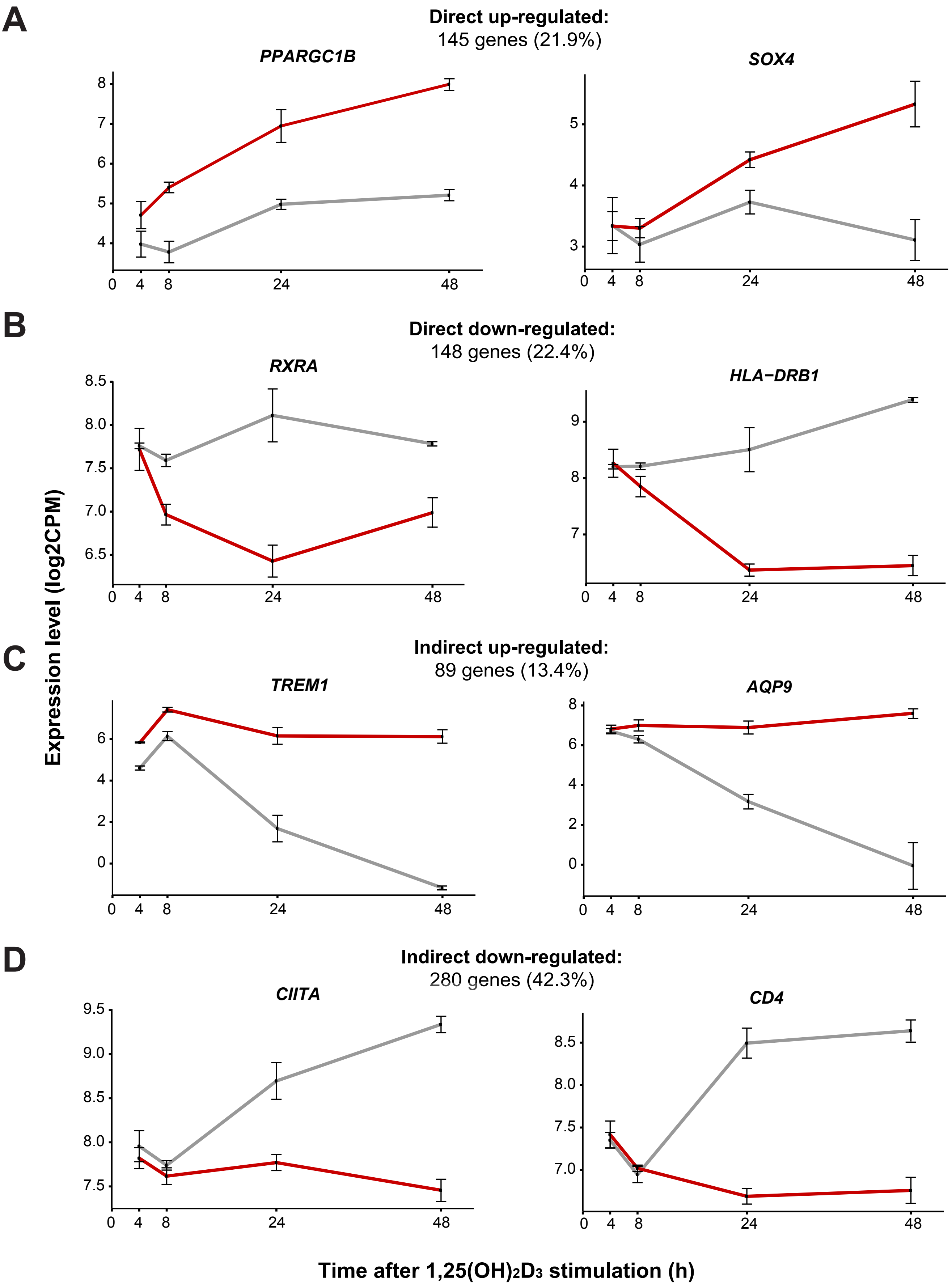
A
Direct up-regulated:
145 genes (21.9%)
PPARGC1B
SOX4
B
Direct down-regulated:
148 genes (22.4%)
RXRA
HLA−DRB1
C
Indirect up-regulated:
89 genes (13.4%)
TREM1
AQP9
D
Indirect down-regulated:
280 genes (42.3%)
CIITA
CD4
Expression level (log2CPM)
Time after 1,25(OH)2D3 stimulation (h)

Fig. S5

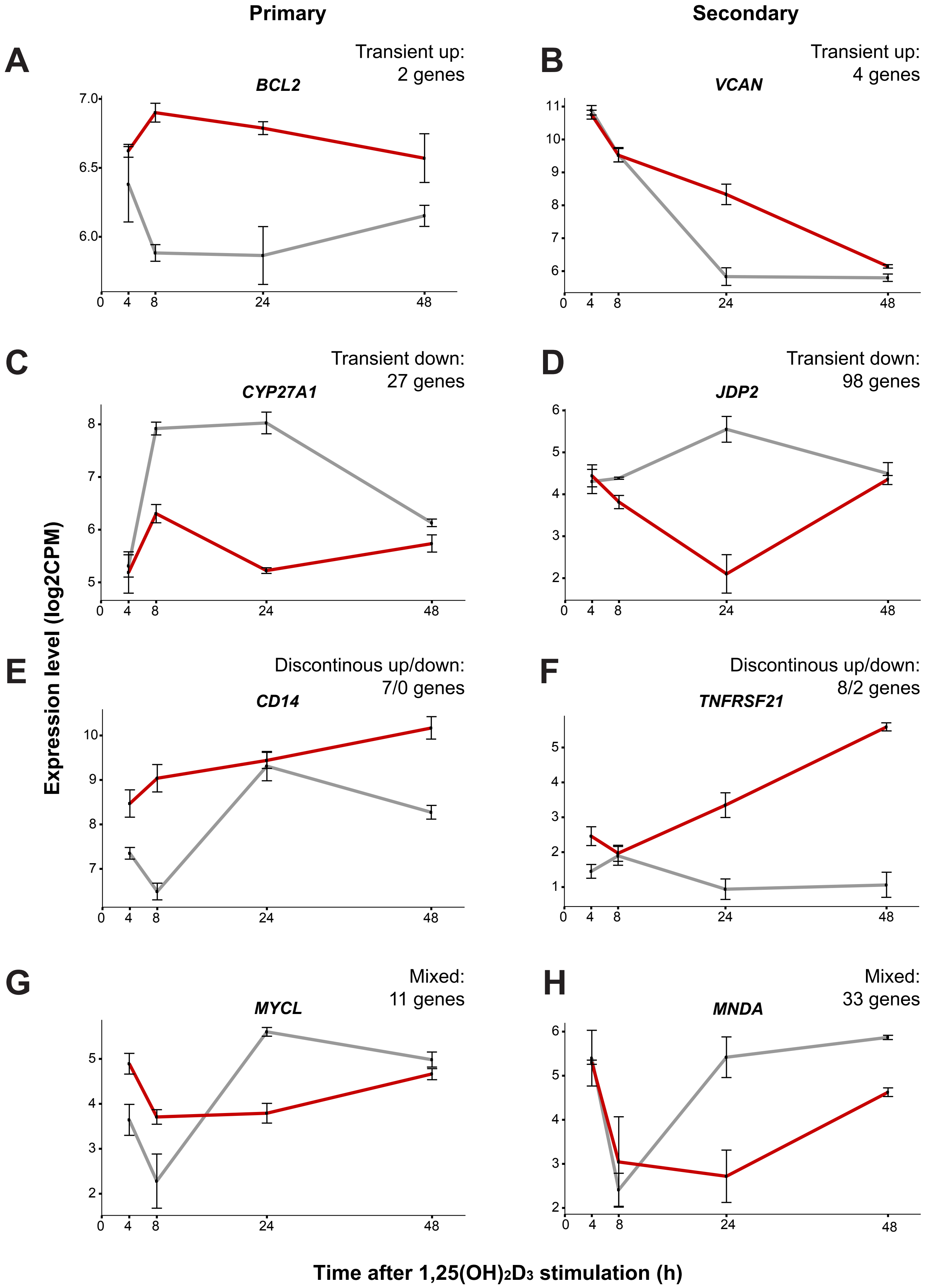
Primary
Secondary
A
Transient up:
2 genes
BCL2
B
Transient up:
4 genes
VCAN
C
Transient down:
27 genes
CYP27A1
D
Transient down:
98 genes
JDP2
E
Discontinous up/down:
7/0 genes
CD14
F
Discontinous up/down:
8/2 genes
TNFRSF21
G
Mixed:
11 genes
MYCL
H
Mixed:
33 genes
MNDA
Expression level (log2CPM)
Time after 1,25(OH)2D3 stimulation (h)

Fig. S6

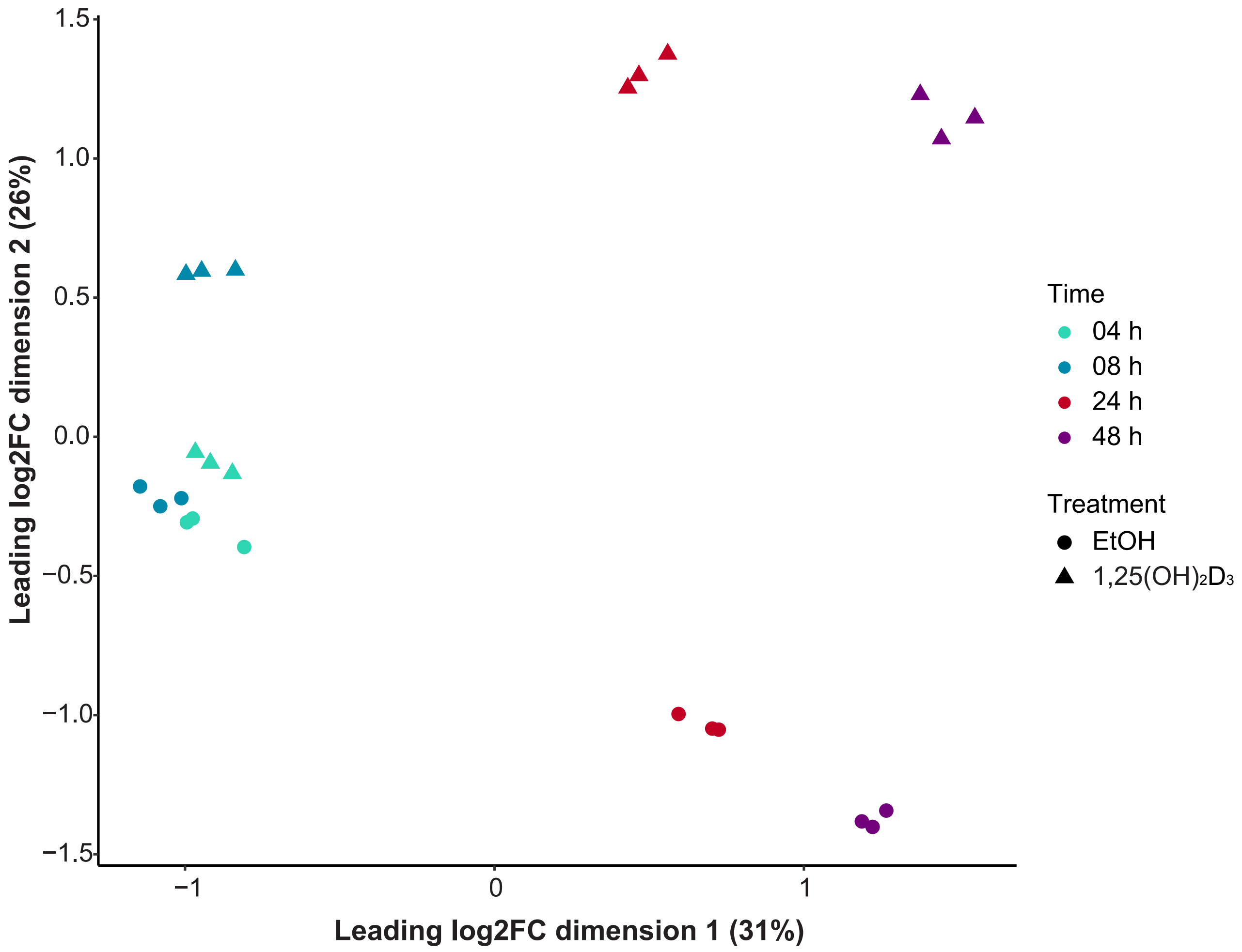

1.5
1.0
0.5
0.0
−0.5
−1.0
−1.5
−1
0
1
Leading log2FC dimension 2 (26%)
Leading log2FC dimension 1 (31%)
Time
04 h
08 h
24 h
48 h
Treatment
EtOH
1,25(OH)2D3

Fig. S7

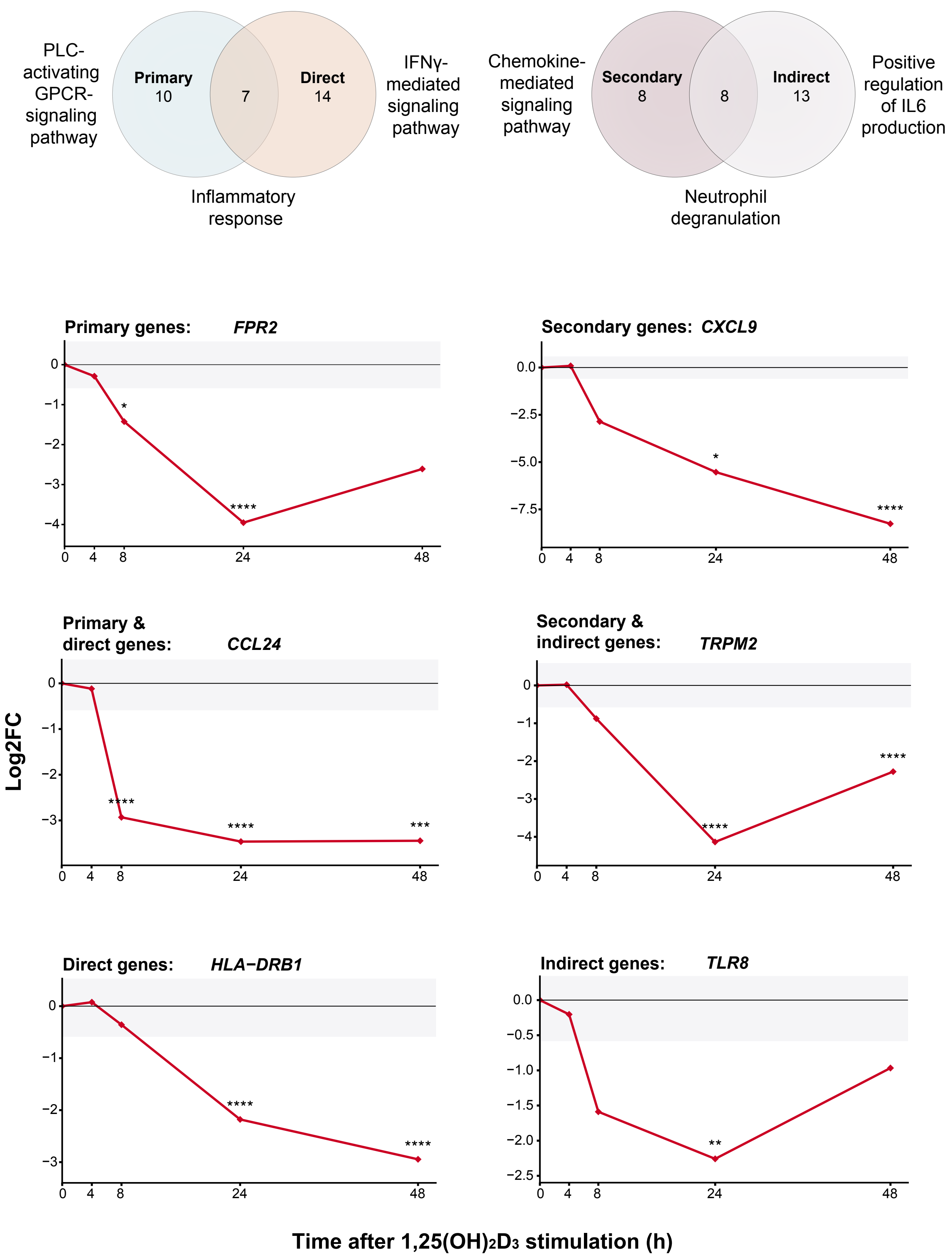
PLC-activating GPCR-signaling pathway
Primary 10
7
Direct 14
IFNγ-mediated signaling pathway
Inflammatory response
Chemokine-mediated signaling pathway
Secondary 8
8
Indirect 13
Positive regulation of IL6 production
Neutrophil degranulation
Primary genes: FPR2
Secondary genes: CXCL9
Primary & direct genes: CCL24
Secondary & indirect genes: TRPM2
Direct genes: HLA−DRB1
Indirect genes: TLR8
Log2FC
Time after 1,25(OH)2D3 stimulation (h)

Fig. S8

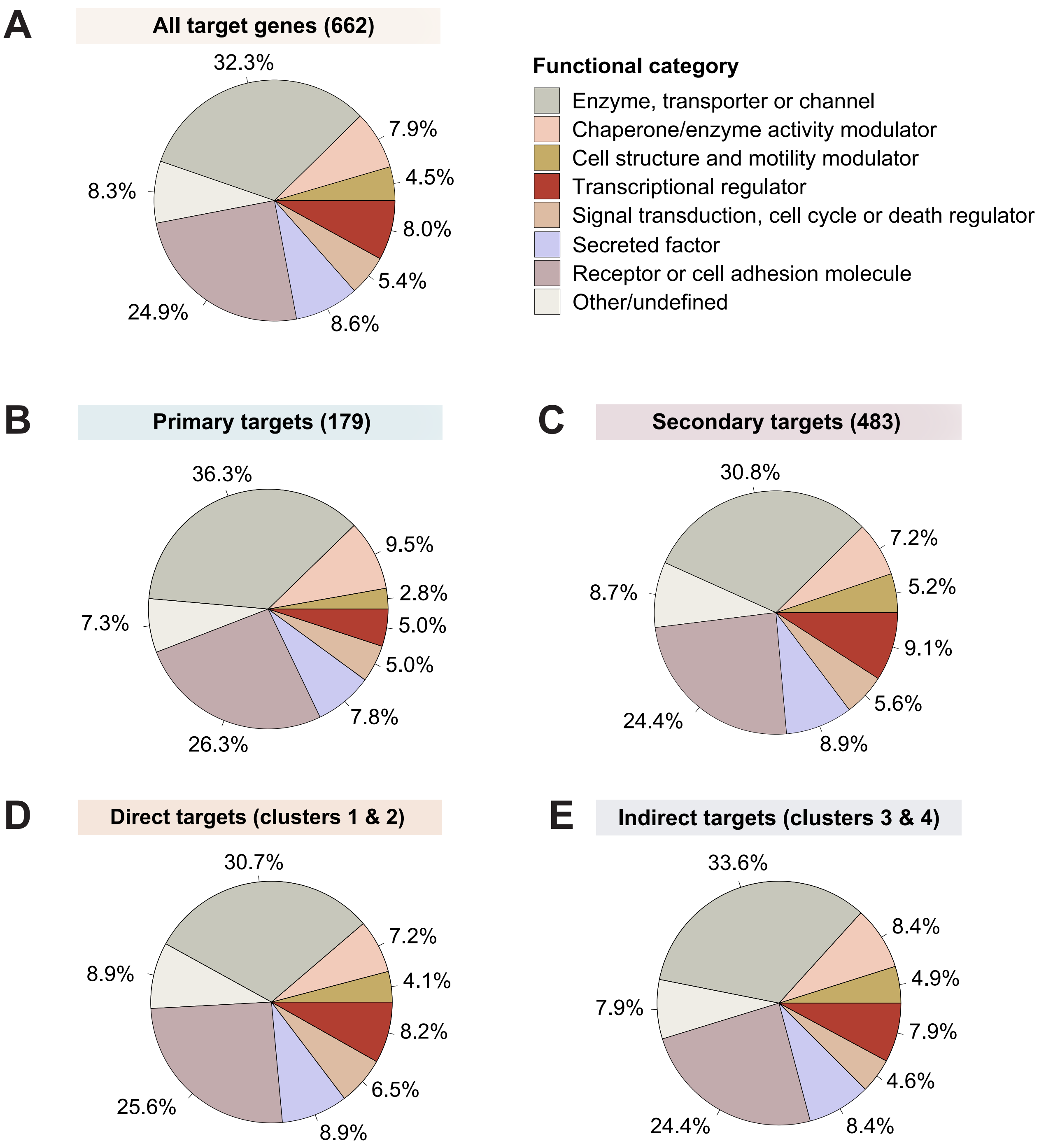
A
All target genes (662)
32.3%
7.9%
4.5%
8.0%
5.4%
8.6%
24.9%
8.3%
Functional category
Enzyme, transporter or channel
Chaperone/enzyme activity modulator
Cell structure and motility modulator
Transcriptional regulator
Signal transduction, cell cycle or death regulator
Secreted factor
Receptor or cell adhesion molecule
Other/undefined
B
Primary targets (179)
36.3%
9.5%
2.8%
5.0%
5.0%
7.8%
26.3%
7.3%
C
Secondary targets (483)
30.8%
7.2%
5.2%
9.1%
5.6%
8.9%
24.4%
8.7%
D
Direct targets (clusters 1 & 2)
30.7%
7.2%
4.1%
8.2%
6.5%
8.9%
25.6%
8.9%
E
Indirect targets (clusters 3 & 4)
33.6%
8.4%
4.9%
7.9%
4.6%
8.4%
24.4%
7.9%
